# Proceedings American Medical Association

**Published:** 1865-07

**Authors:** 


### MISCELLANEOUS.

### PROCEEDINGS AMERICAN MEDICAL ASSOCIATION.

Dr. N. S. Davis, of Chicago, President of the Association, delivered his annual address, as follows:

GENTLEMEN OF THE AMERICAN MEDICAL ASSOCIATION:

In entering upon the discharge of those duties imposed on me by your too generous partiality, one short year since, I was constrained to do it with expressions of deep regret, that the great struggle for subduing a gigantic rebellion was still continuing; and that in consequence, the seats of many of our professional brethren whose cordial hands and warm hearts had so often greeted us, were still vacant. Those expressions of regret were accompanied by the hope, that before the day for this annual gathering should come, the dark and desolating cloud of war would be broken, and give place to the radiant bow of peace, with former friendships restored and our national union unbroken. It is my highest pleasure to congratulate you, to-day, that what we then so fondly hoped for, is now substantially accomplished. The cherished flag of our country again waves in triumph over every part of our almost boundless domain; and the patriotic legions who have borne it, proudly, on so many bloody fields of human strife, are returning to their peaceful firesides, decorated with wreaths of victory and enshrined in a nation's gratitude.

But our congratulations, to-day, are still mingled with a deep shade of sadness. Sadness, that so many of our countrymen have been compelled to sacrifice their lives in defence of the integrity and perpetuity of our government; sadness, that so many of our professional brethren have been constrained to abandon the peaceful pursuit of their humane calling at home, and sacrifice comfort, health and sometimes life, in the noble effort to mitigate the calamities and sufferings of war; and a deeper, more enduring sadness, that to the desperate wickedness of treason, has been added the darkest crime that can disgrace human nature, the deliberate murder of the Chief Magistrate of this great Republic. Let us hope, however, that in this act, the climax of human wickedness has been reached; that the cup of our national calamities has been drunk to its bitterest dregs; and with becoming humility, in the true spirit of our humane calling, let us implore the Sovereign Ruler of the Universe to make our re-union one of hearts as well as States; and our great nation, one in which labor shall everywhere receive its just reward, whether in the workshops and humble cottages of the North or on the sunny plantations of the South.

By a natural transition, the mind turns from these reflections to the work of death in our own ranks, since our last annual interchange of greetings. A few months since, one who has filled the highest position in the gift of this Association, with unrivalled ability, and whose professional skill, ripe scholarship, and noble christian deportment, had endeared him to us all, was called upon to cease his earthly toil and enter upon a higher and holier existence. And at the very hour when our profession was bowed in full sympathy with the national grief for the loss of its Chief Magistrate, our cup of affliction was made to overflow afresh, by the final departure of one, who, by universal consent, had occupied for many years the highest position, especially in the surgical department of our profession, not only in America but throughout the civilized world. Need I mention the names of Jonathan Knight of New Haven, and Valentine Mott of New York? After long lives, ardently and successfully devoted to the dearest interests of humanity, full of years and full of honors, peacefully they have gone to their eternal rest. But their names, their works, and their noble examples are left to us and the generations that will follow. Nor has the work of the destroyer been limited to these; for within a few months past Thomas D. Mitchell of Philadelphia, William E. Coale of Boston, and Sylvester D. Willard of Albany, all members of this Association, and eminent in the profession, have been released from their earthly labors.

With this slight and imperfect tribute to the memory of those whom we shall see no more in our midst, permit me to occupy your attention with some reflections upon the past history, present organization, and future prospects of this great National Medical Association. In submitting these reflections, I shall assume neither the character of an eulogist nor a critic, but shall simply endeavor to draw such lessons from the actual results of the past, as will aid in the discharge of the duties of the present, that still greater benefits may be reaped in the future.

Twenty years have now elapsed since the Medical Society of the State of New York issued the call for the National Convention, from which our present Association had its direct origin. During that period of time, large, pleasant and harmonious meetings have been held in almost every section of our widely extended country, and both time and opportunity have been afforded for developing the actual interests and influences involved in our organization. It requires only a brief examination of our past career, to show that both in the principles of our organization, and in the practical results of our annual meetings, three important interests are directly involved; namely, the improvement of our system of medical education, the direct advance of medical science and practice, and the promotion of social intercourse and fraternal feeling throughout the entire profession. And the thoughtful attendant upon our meetings has not failed to observe, that each of these interests has uniformly attracted a due share of representatives. Indeed, most of the embarrassments attendant upon our past meetings, and the criticisms to which the Association has been subjected, have arisen from the difficulty of accommodating interests so important and varied, in such a manner as to satisfy the advocates of each, in the very brief time hitherto allotted to our annual meetings. Thus, whenever medical education became the theme of discussion, those more interested in the reading and discussion of papers and reports of a direct scientific and practical character, were ever ready to restrict debate, refer the subject to committees, or in some other way avoid what they regarded as a mere waste of time. On the other hand, if the reading of an elaborate scientific paper was commenced, the author would seldom complete the first half dozen pages before the zealous advocate of educational reform would dispose of the whole subject by a motion to dispense with further reading and refer the document directly to the Committee on Publication. While the good-natured lovers of good dinners and sight-seeing would always be ready to aid the other parties with their votes, and to secure the acceptance of every invitation to an entertainment, an excursion, or a public institution.

Under such circumstances, our records soon became cumbered with a multitude of reports and resolutions concerning medical education, and the annual volume of Transactions plethoric with reports and papers that were read to the Association by their titles only; while the social entertainments reached a magnificence and costliness seldom equalled in any other relation of society.

The embarrassments felt from these circumstances led to frequent propositions to change either the constitution of the Association or its rule of business.

To avoid lengthy reports on general topics, all the standing committees on the different departments of medical science were first abolished, and a list of committees on special subjects substituted in their place.

To further economize time, an order was next adopted requiring every report and paper covering more than ten pages of manuscript, to be presented by a brief abstract merely setting forth its title and contents.

To check the evils of extravagant and costly entertainments, the Committee of Arrangements were instructed to omit all such from the programme of arrangements for all subsequent annual meetings.

The practical results of these changes were by no means satisfactory. The first led to the annual appointment of a long list of committees on special subjects, not one in six of whom ever furnished a report of any kind. The second speedily developed the practice of presenting reports and papers by their titles only, and often accompanied by the acknowledgment that they were still unfinished, followed by a vote that they be referred directly to the Committee of Publication. Thus, in many instances, not only deciding to publish papers of which the Association was profoundly ignorant, but which, at the time, had no actual existence except in the minds and memoranda of the authors. The adoption of the third, judiciously designed to check extravagance in social entertainments, has resulted only in exchanging one magnificent public banquet, occupying one evening, for three or four private ones every evening during the annual sessions of the Association. If we add to these considerations the fact that the greater part of the first day of each annual session, embracing one-fourth part of the whole time, has been occupied with preliminary matters of organization, and the election of officers who were immediately required to enter upon the performance of duties for which they were often wholly unprepared, we shall readily perceive the reasons why the practical working of the Association, thus far, has not fully realized the wishes and expectations of many who have labored efficiently for its organization and support.

Some, who entered upon the work with the hope that the Association would be the agency for speedily securing the adoption of a uniform and elevated standard of medical education by all the medical schools of our country, and having in their own minds some favorite plan by which it was to be accomplished, having seen year after year pass without its adoption, very naturally conclude, from their stand-point of observation, that the Association is a failure.

Others, who entered with equal zeal upon the work of organization, with the hope that it would be the means of establishing in the profession of our country a spirit of original scientific investigation, a more complete elucidation of the causes and laws governing the prevalence of zymotic and epidemic diseases, and a higher standard of medical literature, have found, in our short annual sessions and the little attention actually given to scientific and original papers, comparatively little to sustain their zeal, or to deter them from writing over our portals the word *failure*.

Therefore, it is not strange that we should meet here and there an unfriendly criticism, or the uncomplimentary remark that our annual meetings partake more of the character of "gormandizing and sight-seeing," than of grave scientific and professional inquiry; or in casting our eyes over the assemblages of the last three or four years, we should miss the presence of some who contributed so much to the interest and value of all our earlier meetings. But with a frank admission of the defects and embarrassments in the past practical working of this Association, does the conclusion legitimately follow that it has made so little progress in the accomplishment of the important purposes for which it was organized, as to demonstrate its inutility, or to create a well-founded doubt of its ultimate entire success?

To answer this question without prejudice, it is necessary to keep in mind the distinction between the accomplishment of a given purpose and the particular modes or plans by which it is to be attained. Many of the latter may be tried and fail, and the object sought may nevertheless be fully accomplished. It is probable that three-fourths of those who originally looked confidently to this Association as the instrument for elevating the standard of medical education in this country, entertained the idea that it would be effected by some uniform plan embracing better preliminary education, longer lecture terms, more extensive clinical instruction, and more rigid examinations, to be formally adopted by the schools. And inasmuch as no such plan has yet been adopted, it is quite natural that those who had been regarding this as the only method for gaining their purpose, should regard the purpose itself as a failure. And yet such an inference would neither be a logical deduction from the premises, nor in accordance with the actual facts as they exist at the present time.

This will be fully apparent to any one who will compare the number, location and requirements of the medical schools of 1845 with those of 1865. At the former period from 13 to 16 weeks was almost universally adopted as the length of the annual lecture term. Now, the number of colleges in which the lecture term is less than eighteen weeks, are few and unimportant, while some have extended it to five, and others to six months. Then, the number of chairs, or Professorships, was five and six, leaving the important branches of Organic Chemistry, Microscopic Anatomy and General Pathology entirely out, and usually making Physiology only an appendage to the chair of Anatomy. Now, the medical college that does not include all these in its curriculum, would be universally considered as behind the age. At the former period three-fourths of all the medical schools in this country were so located that their students could have no access, whatever, to any true clinical instruction at the bedside of the sick. And in those located at Boston, New York, Philadelphia, Cincinnati and New Orleans, where adequate hospitals existed, the actual clinical instruction was generally limited to one or two visits to the hospitals each week. Now, this is so far reversed, that two-thirds of the whole number of our medical colleges provide some amount of true hospital clinical instruction, and three of them at least are so directly connected with important public hospitals, that their courses of clinical instruction, in all the departments of practical medicine and surgery, are as full and as extensive as any of the other departments of medical science.

The extent and importance of the change in regard to clinicas instruction will be rendered still more apparent by another mode of comparison. For instance, at the time of the organization of this Association, not only three-fourths of our medical schooll were so located as to afford their students no access to hospitals, but a large majority of the whole number of students resorted to them for instruction. Thus, in the New England States there were, at least, six medical schools, of which only the one in Boston was located within reach of an adequate hospital; and yet its classes were often out-numbered by those of Pittsfield. While at the present time, if I remember correctly, the number of students receiving instruction in Boston with access to the Massachusetts General Hospital is nearly, if not quite, equal to that in all the other New England States put together. The time is fresh in my memory, when the number of students annually assembled in a medical college, located on the bleak hills of Herkimer county, in the State of New York, fully equalled those resorting to the schools directly in the great metropolis of that State. Now I think there is not a medical college maintaining an active existence in that State, which does not provide for its students access to a public hospital for clinical instruction.

What is true of New England and New York, in this respect, is equally true of the whole country.

When we remember, that of all the improvements in medical education, demanded by this Association from its primary organization to the present time, none were more prominent, clearly defined, or persistently urged, than an increase in the number of Professorships with an extension of the curriculum; a lengthening of the annual lecture term; and the addition of full hospital clinical instruction both in practical medicine and surgery; we see how closely the improvements actually made correspond with the demands of the Association, and how nearly the objects sought have been already accomplished. It is true that they have not been accomplished by the formal adoption of any particular plan or concert of action, and perhaps not wholly through the influence of this Association; but that their accomplishment has resulted mainly from the strong concentration and persistent expression of public sentiment through this Association, there can be no reasonable doubt.

Neither has the influence of our organization upon the progress of medical science and literature been as feeble as many suppose. At the commencement of our associate existence, the number of original American medical works was comparatively small; and the universal complaint was, not only that American talent was spent only in the editing of foreign books, but that our own writers, even of the highest reputation, found it extremely difficult to find publishing houses willing to issue their works from the press. The able reports on this subject and on medical literature made to the earlier meetings of this Association, and the consequent general awakening of attention to it, throughout the whole profession, has resulted in so completely reversing the former state, that we now rarely see the name of an American writer appended merely as editor of a foreign work; while the medical press of our country teems with original medical works of high merit in every department of medical science. And not only so, but the shelves of the laborious practitioners of our humane art, throughout the whole country, now contains three American to one foreign work, especially in the departments of practical medicine, surgery and obstetrics. And whoever examines the series of published Transactions of this Association, will not only find a number of essays, which for scientific merit would do credit to the investigators of any other country, but they will find much additional evidence that attention has been directed to most important inquiries concerning the causes and prevalence of epidemic diseases; the influence of topography and climate on endemics; and the nature and therapeutic value of indigenous articles of the materia medica, not only among our own members, but also throughout many of the State, county, and distant medical societies in every part of the country.

Socially, the success of our organization has been all that the most ardent could desire. It has not only removed local prejudices and sectional jealousies, but it has awakened everywhere the most liberal hospitality and the most cordial friendships. It has, indeed, made neighbors and friends of families whose homes are a thousand miles apart; while it has infused new life into many old State and local societies, and stimulated the profession to the formation of many new ones.

From a deliberate and candid examination of the whole past history of the Association, with a full appreciation of the embarrassments arising from short annual sessions, and imperfect arrangements for the transaction of business, I am fully satisfied that so far from having proved a failure, it has made such substantial progress in the accomplishment of every important object of its creation, as to afford the fullest assurance of its final complete success. Hence instead of entertaining doubts, or yielding to feelings of hesitation or discouragement, every friend of the social organization of the profession, and every advocate of advancement in its educational, scientific and literary interests, should give it his most cordial support. Instead of abandoning the work of twenty years past, merely because it is not yet perfect, true wisdom would dictate the careful removal of such hindrances and imperfections as time and experience had developed, that the great and important work, itself, might be pushed more rapidly to completion.

Having carefully and anxiously watched the progress of this Association, from the incipient steps of its organization to the present time, I trust those assembled on this occasion will pardon me if I devote the remainder of this address to a brief and explicit statement of those measures, which seem to me sufficient, if judiciously executed, to ensure its complete success and perpetuity.

Although it is apparent that most of the evils and embarrassments which have attended our past meetings have arisen from an attempt to crowd a consideration of the educational, scientific and social interests of the profession into the short space of three or four days, yet it is by no means desirable to abandon either of these interests in the future. The exact and all-important desideratum needed at this stage of our progress, is such an apportionment of time to each of these interests as their relative importance demands; and the time allotted to each so systematically used as to develop the highest degree of efficiency in the results. To have a time and place for each legitimate interest, and to keep each in its place, is a matter of permanent importance in an organization so extensive as ours. Happily, the arrangement for attending to the scientific interests of the Association in sections, first carried into effect in 1860, and the amendments to the constitution adopted in 1864, have removed all impediments to the adoption of a most complete and efficient plan of operations at each annual meeting. Let the morning sessions of moderate length be devoted to the general business of the Association and the consideration of all matters relating to medical education, together with the simple presentation of all scientific reports and papers by their titles, that each may be referred to the section most appropriate for its consideration. Let all the afternoons and evenings, except one evening of each session, be set apart exclusively for the consideration of the scientific interests of the Association in the capacity of sections. This would leave only the one evening of each session to be devoted, in a formal manner, to purely social interests. To some, this limited time may seem insufficient. But if we remember that all such as are more interested in sight-seeing and mere social intercourse than in the advancement of science and literature, can gratify their preferences at any part of the meeting without interrupting either the general sessions or the sections, it will be conceded that the time specified is quite as much as the *relative* importance of the interest to be served requires.

But the arrangements for that evening should be such as would permit the most free and cordial intercourse. A public hall should be provided in which gentlemen and ladies could mingle and promenade freely; where each could seek out his old friends and make the acquaintance of new ones; where wit, repartee, and if need be songs, sentiments and speeches could be made to enliven the evening. A simple stand might be placed in some corner, where all who wished could obtain a dish of ice-cream, strawberries or other fruit, and a cup of coffee. But there should be neither ostentatious show, nor rich viands, nor strong drinks, for the acknowledged guardians of the public health should not, especially in their highest representative capacity, themselves publicly violate the plainest laws of hygiene.

That part of our proceedings which has been the subject of most serious complaint, during the last few years, has related to the consideration and final disposition of such reports and papers as related to the scientific and practical interests of the profession. And it may appear to many that the time which has been indicated as proper to devote to those topics is still wholly inadequate for their proper examination and disposition.

If, however, all such papers and reports are called for and referred to the appropriate sections, before the close of the first morning session, as they certainly should be, it is confidently believed that adequate attention could be secured for every subject properly presented for consideration. If the order of business is so arranged as to accomplish this, the several sections can commence their work on the afternoon of the first day of each annual meeting, thereby securing from three to four full afternoons and evenings for their important work; and if all the sections are properly organized and the subjects for consideration judiciously distributed among them, it will be equivalent in practice to a multiplication of these three or four afternoons and evenings by six; or a practical extension of the time devoted to the scientific interest of the Association to three or four weeks.

And in this connection I wish to call your serious attention to the more complete and efficient organization of the sub-divisions of the Association. As each section is authorized to choose its own officers and adopt its own rules of action, their existence should, by no means, be regarded as ended at the adjournment of each annual session. But a President and thoroughly qualified Secretary should be chosen for the entire year; and each should adopt a series of well considered rules which should govern the reading, discussion, and final disposition of all reports, papers and questions that legitimately come before it. Among the rules thus adopted, should be one, requiring every report and paper to be so far complete at the time of its presentation, that if deemed worthy of publication it can be passed from the custody of the section directly to the Permanent Secretary, without being detained by the author for either revision or completion. Another should peremptorily forbid the reference of any report or paper on a scientific or practical subject, to the Committee of Publication, until the same has been sufficiently examined by the section, to know its length, its actual contents, and the number and character of the illustrations, if any, that are to accompany it. If there should happen to be more papers referred to any one section, than could be fairly examined during the time the Association is in session, such surplus papers should be referred to judiciously selected sub_ committees, with instructions to complete their examination and report on the same to the Permanent Secretary within thirty days after the adjournment.

The adoption of such rules, and the rigid adherence of each section to them, would accomplish two very important objects. First, it would more effectually guard against burdening the Association with the publication of matters appropriate only for the pages of an ordinary medical periodical, and would secure the Committee of Publication against unnecessary delays in the reception of such matter as should be actually designed for publication in the Transactions of each year. Second, by rendering it certain that every report or paper properly prepared and presented in time, would receive a fair hearing and consideration, a very much larger number of writers and investigators of ability would be induced annually to present the products of their labor for the consideration of the Association.

Permit me to make one more suggestion in relation to the sections, namely, that each section should be provided with either a skillful secretary or a professional reporter, who in addition to the simple record of proceedings, should preserve a correct summary of all discussions on scientific questions and papers, and report the same to the Permanent Secretary, that so much of it as was of importance could be published in connection with the papers to which it might relate, in the Transactions of the Association. This would not only preserve many valuable facts and observations in a small compass, but it would present a strong additional inducement for the most active and experienced minds in the profession to attend and participate in the doings of the sections.

I hope the foregoing suggestions will be regarded as worthy of a prompt and careful consideration by the several sections of our present session.

I would also suggest to the Association the propriety of dispensing with the appointment of a long list of special committees annually, which seems to have served little other purpose than to advertise the names of those receiving the appointment; and instead thereof, allow each section annually to select such subjects for investigation, and appoint such committees to investigate them, as they may deem most profitable. This need not interfere in the least with the reception of voluntary communications on any subject, in the same manner as at present; and yet I am confident it would insure the selection of more important topics; cause them to be more equally distributed among all the important branches of medical science, and secure their more prompt and thorough investigation.

It should be a leading object of the scientific department of our Association, to awaken and foster in the profession an active spirit of experimental research and of rigid deductive investigations. It should also exert an important influence in directing such researches into the most profitable channels, by more carefully selecting the most appropriate topics for investigation from year to year.

If each section now provided for by our by-laws would perfect its organization and designate two or three important topics for investigation the coming year, it is certain that it would give to the scientific interests of this Association a scope and efficiency far superior to the present or any previous methods of procedure. It has also appeared to me that such a change might be made in the mode of disposing of the papers presented to this Association, as would liberally encourage contributions and yet greatly increase the scientific character of our annual volume of Transactions. According to my views, the volume of Transactions published to the world, by such an Association as this, should contain no papers except such as embody a complete deductive review of the topics discussed, developing and establishing important rules of practice; or the results of such well-devised series of experiments or observations as clearly indicate a positive addition to our stock of knowledge concerning some one of the departments of medical science. Such papers and reports as might be presented and referred to the several sections, which though neither complete as deductive essays, nor clearly establishing new facts, yet containing fragmentary items or cases of value, or suggestions worthy of further investigation, should be recommended for publication in such regular medical periodicals as their authors might choose; while such, only, as were found worthless should be returned to their authors.

It might be feared by some, that the adoption of such a rule of proceeding would make our annual volume of Transactions very small. Be it so. It certainly is not the *bulk* of the volume, but the *quality* of the matter it contains, which is to affect both our reputation and our usefulness. Perhaps one of the most important topics connected with medical science, which still needs elucidation, is the connection of atmospheric conditions with the prevalence of certain forms of disease. But to properly investigate that subject, it is absolutely necessary to have a perfectly reliable register of the Thermometric, Barometric, Hygrometric, Electric and Ozonic conditions of the atmosphere, kept in each important geographical section of our country through a series of at least ten consecutive years, in direct connection with a corresponding record of the prevalence of the various forms of disease in the same localities. And I suggest whether, if the rule just mentioned in regard to the publication of papers in the Transactions, should so far diminish the size of the annual volume as to leave a surplus in the Treasury, it would not be more profitably spent in furnishing the necessary instruments and establishing the necessary records, to determine with accuracy, in a few years, the actual relations of appreciable atmospheric conditions to the prevalence and character of diseases, than in the publication of such papers as serve little other purpose than to increase the size of the volume of Transactions. Indeed, if capable and zealous members of the profession, furnished with the necessary instruments, could be employed in each section of the country, and the results of their observations carefully arranged, tabulated and reported to this Association annually; where, in the section on Meteorology and Epidemics these results could be closely scanned, and have added to them the more desultory observations of the profession generally, it could not fail to throw a flood of light upon the etiology of a large class of most important diseases.

With becoming deference I submit the foregoing suggestions for your consideration. They contemplate no changes in our Constitution or plan of organization; they propose the introduction of no new or untried schemes; but they have for their sole object, the removal of obstructions and objections which the experience of the past has demonstrated to exist, and the development of a more complete systematic and efficient method of transacting all the important business of the Association.

The great object is to perfect and perpetuate what has been, already, so well begun.

This great *National Medical Organization* has already existed long enough to have passed the dangers and uncertainties of its childhood, as well as the fickleness of its youth. It is time that its principles, its modes of action, and its important objects, were clearly defined, methodically arranged, and matured to the steadiness and vigor of early manhood. Many of the most renowned members of our profession, who took part in its organization and watched over its earlier years, have been gathered to the home of their fathers; and the nineteen years of active toil that has been added to the lives of many others, have carried them beyond the period of ardent active labor, to the more quiet era of ripening age. They still mingle with us, and at each returning anniversary meeting we hail their presence and crave their council, with the same joy and reverence, that characterizes the meeting of the filial son and virtuous father.

But the important question whether this Association, which has already accomplished so much for the advancement of the Educational, Scientific and Social interests of our noble profession, and maintained a vigorous and unsullied career during the nineteen eventful years that are past, shall be maintained, its modes of action perfected, and its beneficial influences constantly extended, depends entirely upon the generation who are now in the active, vigorous period of early manhood. If those of this class whom I now see before me, have imbibed the spirit of the founders of this Association, and will come forward with alacrity to the work of sustaining and perfecting what their fathers in the profession have begun, its existence will not only be perpetuated from generation to generation, but its beneficent influences will widen and deepen with every returning anniversary. Candor compels me to admonish you, however, that such a result can never be accomplished by any amount, either of good *wishes* or *fault-finding;* but by prompt, persevering, disinterested action. Let all those who desire to see the standard of medical education steadily elevated from year to year, continue to concentrate, and give expression to, public sentiment through the morning sessions of each anniversary meeting. Let all those who have been complaining for years past, that sufficient attention has not been given by the Association to scientific matters, appear promptly in the several section rooms this afternoon, and aid in organizing and putting into efficient operation each of those sub-divisions of our organization. The very complaints and criticisms in which you have heretofore indulged, have demonstrated your appreciation of the work to be accomplished. Hence I feel the more freedom in cordially inviting you to an active participation in the good work.

If the generation, into whose hands are now passing the labors, the honors, and the responsibilities of our time-honored and most beneficent profession, will give faithful heed to these things, the American Medical Association will not only outlive whatever changes and convulsions may be in store for our loved country in the future, but its members will annually come up from the North, the South, the East and the West, to sit in social harmony, and plan additional means for alleviating human suffering, so long as civilization itself shall continue to bless the tribes of earth.—Finally, let us all remember, not only while transacting the business of this Annual Session, but also in all the work that is before us in the future, that the great object of a virtuous and happy life, is neither worldly honors nor worldly treasures, but an inward consciousness of doing GOOD from day to day.

